# Endospore-forming *Bacillus subtilis* isolated from third molar exudates and its association with cardiovascular disease: a retrospective cohort study

**DOI:** 10.3389/froh.2025.1726295

**Published:** 2026-01-23

**Authors:** Nadia Jebril, Shahlaa Chabuk, Aseel Al-Sabary, Nibras Al-Mansouri, Worood Al-Jobouri, Samar Al-Saidi

**Affiliations:** 1 Department of Biology, College of Sciences for Women, University of Babylon, Babylon, Iraq; 2 Department of Medical Physiology, Hammurabi College of Medicine, University of Babylon, Babylon, Iraq; 3 College of Medicine, Al-Mustaqbal University, Babylon, Iraq; 4 Department of Pathological Analysis, College of Science, Al-Qasim Green University, Babylon, Iraq

**Keywords:** cardiovascular disease, cohort study, endospore-forming *Bacillus subtilis*, third molar

## Abstract

**Background:**

Various techniques have been previously modified to reduce early postoperative complications following third molar extraction. Given the influence of the oral microbiome, increasingly resistant bacteria have been linked to systemic diseases such as cardiovascular disease (CVD).

**Objective:**

In this study, we aimed to identify the spore-forming bacterium *Bacillus subtilis* isolated from third molar exudates and to investigate its potential association with CVD.

**Methods:**

In Iraq, dental hospitals don't keep thorough medical records for each patient. This lack of documentation makes it tough to carry out hospital-based research. Therefore, by collecting exudates from third molars, we conducted a retrospective cohort study of the population undergoing third molar exudate removal in a private dental clinic as an alternative setting to compare cardiovascular outcomes between individuals with cardiovascular disease and controls. Based on clinical assessments, body mass index, LDL cholesterol, C-reactive protein, systolic blood pressure, diastolic blood pressure, hypertension, and smoking status were measured. The study was conducted on 40 men, comprising 20 patients with diagnosed cardiovascular disease and 20 controls. Light and transmission electron microscopy were used to perform a phenotypic evaluation of the bacterial isolates (spore formation, biofilm production). Biofilm formation was assessed using Congo red agar, crystal violet staining, and scanning electron microscopy (SEM). In addition, systolic and diastolic blood pressure (SBP and DBP) values were obtained to further assess cardiovascular risk.

**Results:**

The number of *B. subtilis* isolates was higher in the CVD group than in the control group (non-CVD) and demonstrated significantly greater biofilm-forming ability (OD_600_ = 1.45 ± 0.22 vs 0.85 ± 0.19, *p* < 0.01). TEM confirmed dense endospore architecture from patients with CVD, while SEM revealed extensive extracellular matrix formation within CVD biofilms. Patients with oral colonization by *B. subtilis* showed a significantly higher prevalence of CVD (32.6%) compared to those without colonization (12.6%, *p* = 0.008). The presence of biofilm-positive *B. subtilis* strains was independently associated with CVD (OR 2.91; 95% CI, 1.23–6.83). Spore-forming *B. subtilis* isolates from third molars of patients with CVD demonstrated enhanced biofilm formation and sporulation phenotypes. A moderate positive correlation (*r* = 0.48) was also observed between *B. subtilis* presence and SBP and DBP. These findings suggest that these bacterial characteristics are potentially the cause of systemic inflammation and represent a potential microbial link to CVD.

**Conclusion:**

In countries like Iraq, researchers run into real problems when they try to study links between oral health and other diseases. There's no NHS dental system, so they can't rely on existing records for data. Therefore, this study provides a protocol for conducting investigations related to oral health through collaboration with other institutions, such as universities. With respect to the main finding of this study, spore-forming *B. subtilis* isolated from third molar exudates demonstrated phenotypic characteristics that may contribute to persistent oral colonization and increase systemic inflammatory risk. The observed association with cardiovascular disease warrants further investigation into oral–systemic microbial pathways.

## 1 Introduction

**Figure 1:**
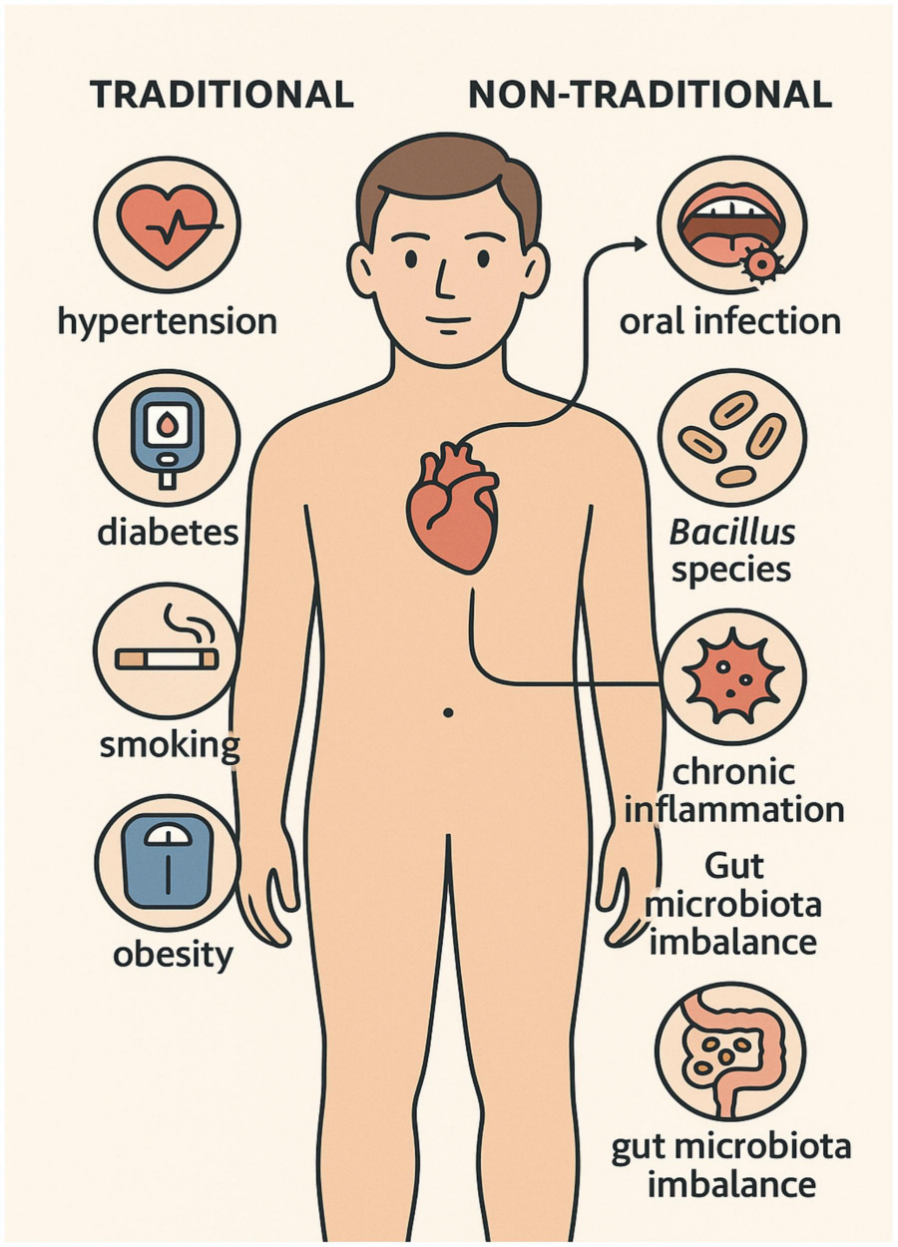
Non-traditional and traditional risk factors of CVD in humans. Icons were sourced from https://www.flaticon.Es.

The human oral cavity harbors a diverse microbial community, comprising up to 700 bacterial species (1). Among these microbes, *Bacillus subtilis*, a Gram-positive, rod-shaped, endospore-forming bacterium, has traditionally been regarded as a non-pathogenic environmental species (2, 3). However, the presence of *Bacillus* species within the human body may contribute to disease development on mucosal surfaces, especially when systemic barriers are compromised (4, 5). Wisdom teeth (third molars) are often extracted due to infection or pericoronitis, and the surrounding tissues frequently show signs of chronic, low-grade inflammation or bacterial colonization (6, 7). In addition, bacterial development may occur as a postoperative complication following third molar extraction (8), mainly emerging approximately 1 month after the procedure (9). Despite standard sterilization procedures and oral hygiene practices, bacteria such as *B. subtilis* can survive in the oral environment due to their structural composition and resistance to antibiotics (10, 11). *B. subtilis* forms spores that, upon germination, can become metabolically active, interact with oral tissues, and transmit to other systems within the human body (12–14). Cardiovascular disease (CVD) remains the main cause of morbidity and mortality. While traditional risk factors, such as hypertension, hyperlipidemia, and smoking (Figure 1), are well established, emerging evidence indicates that chronic bacterial infections, however, oral dysbiosis may contribute to endothelial dysfunction, systemic inflammation, and atherogenesis (15, 16). On the other hand, oral bacteria, including spore-forming species, can play a role in the pathophysiology of CVD, as supported by the detection of microbial DNA and viable bacteria within atherosclerotic plaques (17). In this context, *B. subtilis*, isolated from third molar exudates, may serve as a biomarker of capacity or a contributor to systemic inflammation and cardiovascular risk. Its ability to form resilient spores and its potential to translocate from the oral cavity to systemic sites warrant further investigation into its affiliation with cardiovascular pathology. Retrospective studies focusing on microbial identification from oral resources, combined with corresponding scientific data, can shed light on this underexplored association. This study aims to evaluate the presence of *B. subtilis* in third molar exudate samples and to investigate its potential association with cardiovascular disease within a retrospective cohort framework. The findings may also offer novel insights into the microbial etiology of CVD and provide a greater understanding of oral–systemic health interactions.

## 2 Methods

### 2.1 Participant selection and cardiovascular data diagnosis

**Figure 2:**
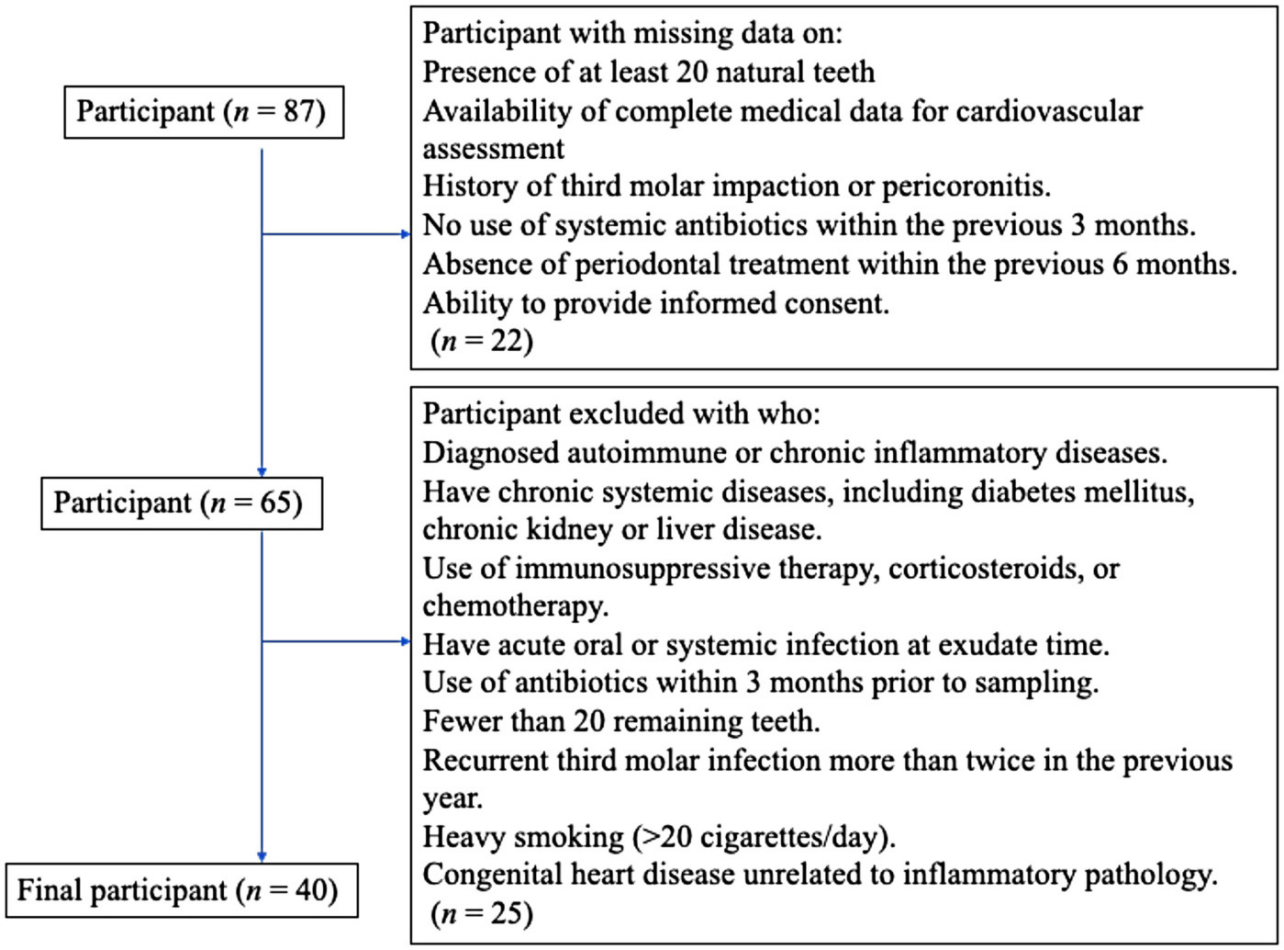
Flowchart of eligibility and exclusion criteria for participant selection.

**Figure 3:**
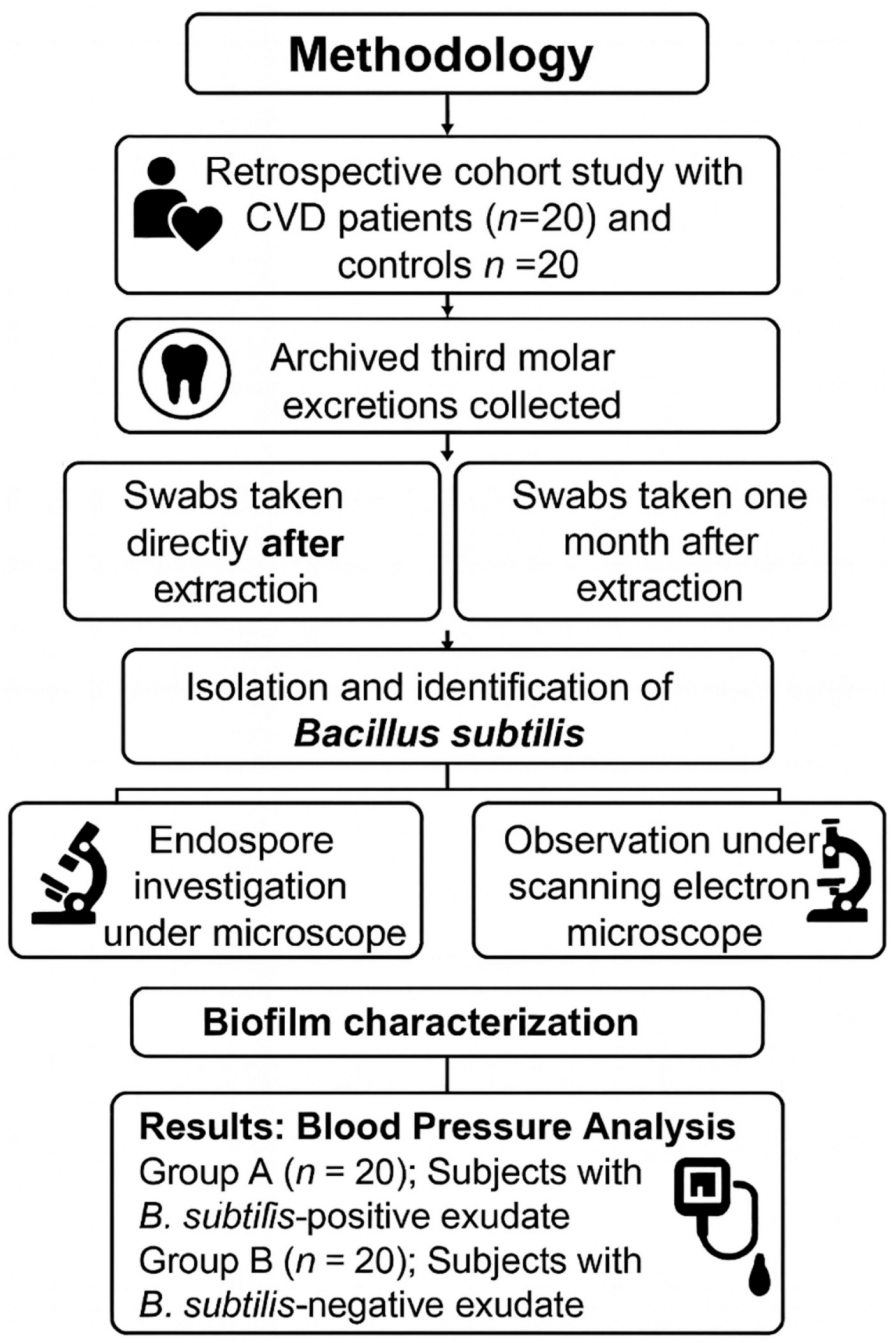
Flowchart of the study design investigating endospore-forming *B. subtilis* isolated from third molar exudates of men diagnosed with CVD (*n* = 20) and men without CVD (*n* = 20) . Icons were sourced from https://www.flaticon.Es.

This retrospective study was conducted on 87 adults aged 25–60 years who underwent third molar extraction at a private dental clinic in 2025. Participants with incomplete data or meeting exclusion criteria were removed from the analysis. As the excluded participants were mainly women, the final retrospective cohort consisted solely of men participants (*n* = 40) (Figure 2). Participants with diabetes, autoimmune disorders, or a history of recent antibiotic use were excluded to minimize microbiome-altering confounders, thus strengthening the reliability of the observed association between *B. subtilis* and cardiovascular risk. The study included two comparison groups: 20 men clinically diagnosed with cardiovascular disease based on blood sample analyses, including measurements of low-density lipoprotein (LDL) and C-reactive protein (CRP), and an additional 20 men who were healthy and free of cardiovascular disease or infections (Figure 3). A questionnaire was designed to confirm a history of third molar impaction in all participants (Table 1). After the teeth were extracted, patients were prescribed antibiotics (amoxicillin 500 mg, three times daily), painkillers, and a mouthwash. Because all patients undergoing third molar extraction exhibited prior signs of impaction or localized inflammation, a control group of non-infected extraction cases was not included in this study. Microbial flora from non-infected cases would present a distinct oral microbial ecology, potentially interfering with the aim of the comparison between CVD and non-CVD groups.

**Table 1:** Demographic summary of 40 men, including patients with CVD (*n* = 20) and men without CVD (*n* = 20).

Characteristic	CVD group (*n* = 20)	Control group (*n* = 20)	*p*-Value
Age (years), mean ± SD	51.2 ± 6.8	49.5 ± 7.2	0.34
BMI (kg/m²)	28.1 ± 3.9	25.6 ± 3.2	0.02*
Smoking status, *n* (%)	8 (40%)	4 (20%)	0.18
Hypertension, *n* (%)	15 (75%)	2 (10%)	<0.001*
LDL (mg/dL)	143.6 ± 18.4	118.2 ± 21.1	<0.001*
CRP (mg/L)	4.2 ± 1.5	1.6 ± 0.8	<0.001*
Systolic BP (mmHg)	144 ± 11	122 ± 9	<0.001*
Diastolic BP (mmHg)	92 ± 7	78 ± 6	<0.001*
Third molar infection history	14 (70%)	11 (55%)	0.32

SD, standard deviation; BMI, body mass index = weight (kg)/height^2^ (m^2^); LDL, low-density lipoprotein; CRP, C-reactive protein; systolic; BP, blood pressure.

* Statistical significance was set at *p* < 0.05.

### 2.2 Ethical approval

In Iraq, the lack of medical records for individual patients in dental hospitals limits the feasibility of hospital-based investigations. By collecting third molar exudate samples from patients undergoing third molar extraction in private dental clinics as alternative settings, it is possible to conduct a retrospective cohort study using oral swabs or blood samples to compare cardiovascular outcomes between affected individuals and controls. Ethical approval was obtained from the College of Science for Women, University of Babylon, Iraq (Approval 64, dated 30 August 2025). Participants were asked to sign a consent form to participate in the study, which included a list of questions detailing the research objective and information to be collected, distributed via Google Sheets. No identifying personal information was recorded.

### 2.3 Collection of swipes samples and identification of *B. subtilis*

*B. subtilis* was isolated immediately and 1 month after extraction to characterize the bacterial isolates at the inflamed surgical site and within the healed exudate area, respectively. Swabs were obtained immediately after third molar extraction from all participants (*n* = 40), including men with CVD and men without CVD. To assess *B. subtilis* colonization after the extraction, additional swabs were obtained 1 month after extraction. Sterile swabs were used to isolate and identify spore-forming *B. subtilis*. Samples were inoculated onto blood agar and nutrient agar plates and incubated at 37°C for 48 h. Colony morphology was examined for identification. Biochemical identification was subsequently confirmed using the IMViC tests, as well as catalase and oxidase assays.

### 2.4 Observation of endospores using light microscopy and TEM

Endospore formation was assessed using the Schaeffer–Fulton method with malachite green to visualize spores and cells (18). TEM was used to visualize intracellular endospores and the spore wall structure after staining with osmium tetroxide, as described by Jebril et al. (19).

### 2.5 Biofilm screening and observation using scanning electron microscopy

Both qualitative and quantitative methods were used to assess biofilm formation in all *B. subtilis* isolates obtained from individuals with and without cardiovascular disease. On *Congo* red agar (CRA), black colonies with a dry, crystalline appearance were indicative of strong biofilm formation (20), as confirmed by the crystal violet assay, which measured biofilm biomass at an optical density of 600 nm (OD_600_). In addition, scanning electron microscopy (SEM) was used to visualize the surface architecture and spatial arrangement of biofilms after fixation with glutaraldehyde and gold plating.

### 2.6 Relationship between *B. subtilis* isolation and CVD

After *B. subtilis* was isolated from swabs of the two study groups (20 patients with CVD and 20 non-CVD controls), the potential association between the number of endospore-forming *B. subtilis* isolates and CVD after third molar extraction was evaluated for each participant.

### 2.7 Relationship between *B. subtilis* isolation and blood pressure

The potential relationship between *B. subtilis* isolated from men diagnosed with cardiovascular disease and their cardiovascular risk was evaluated, alongside systolic and diastolic blood pressure (SBP and DBP) measurements, in 40 men who underwent third molar extraction. Participants were divided into two groups based on the presence or absence of spore-forming *B. subtilis* in oral secretions.

### 2.8 Statistical analysis

SigmaPlot version 14 was used for data visualization. Student's *t*-test and one-way ANOVA were applied to the data, while the chi-square test was used to analyze the number of *B. subtilis* isolates, biofilm formation, and blood pressure, in order to determine associations between third molar exudate findings from individuals diagnosed with cardiovascular disease (CVD) and non-CVD controls. In addition, the chi-square test was used to evaluate the association between CVD status and *B. subtilis* isolation, as these variables were categorical rather than continuous.

## 3 Results and discussion

### 3.1 Description of the study cohort

This retrospective cohort study investigated stored third molar exudate samples from a Babylonian population, comprising 120 swabs collected from 40 men, including 20 patients with CVD (*n* = 20) and 20 men without CVD (*n* = 20). A questionnaire form was designed to confirm a history of third molar infection in all participants (Table 1).

### 3.2 Isolation and identification of *B. subtilis*

**Figure 4:**
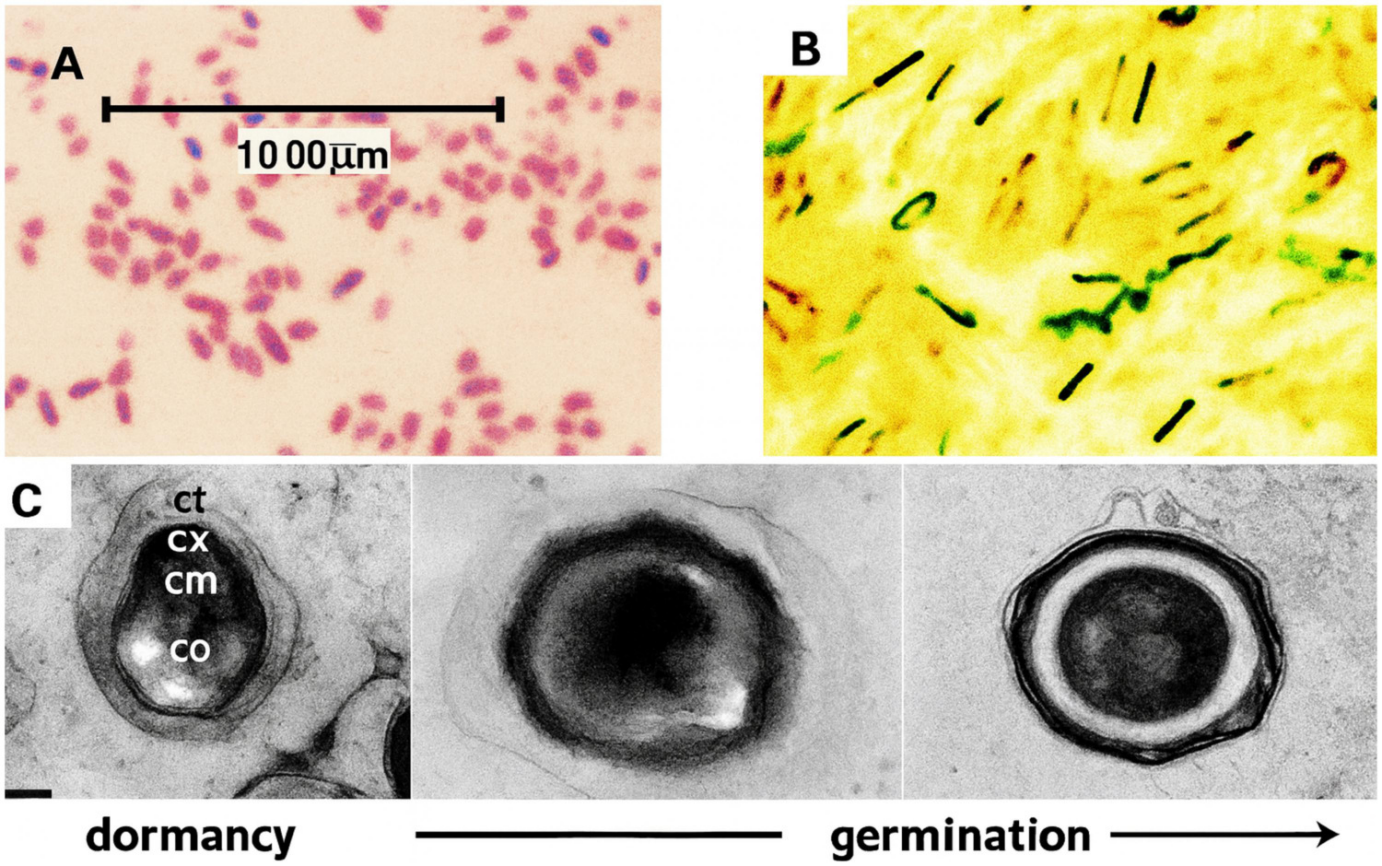
**(A,B)** Optical microscopy images of Gram-positive *B. subtilis* and malachite green endospore staining, respectively, confirming the presence of endospores.Optical microscopy provides lower resolution than TEM. **(C)** TEM image showing detailed ultrastructure, including the cortex, coat, and core, as described by Jebril (20).

Light microscopic examination revealed Gram-positive, rod-shaped, spore-forming bacteria (Figure 4A). Biochemical testing showed that *B. subtilis* isolates were catalase-positive, oxidase-negative, indole-negative, and urease-negative and exhibited motility, allowing them to move in soft agar.

**Figure 5:**
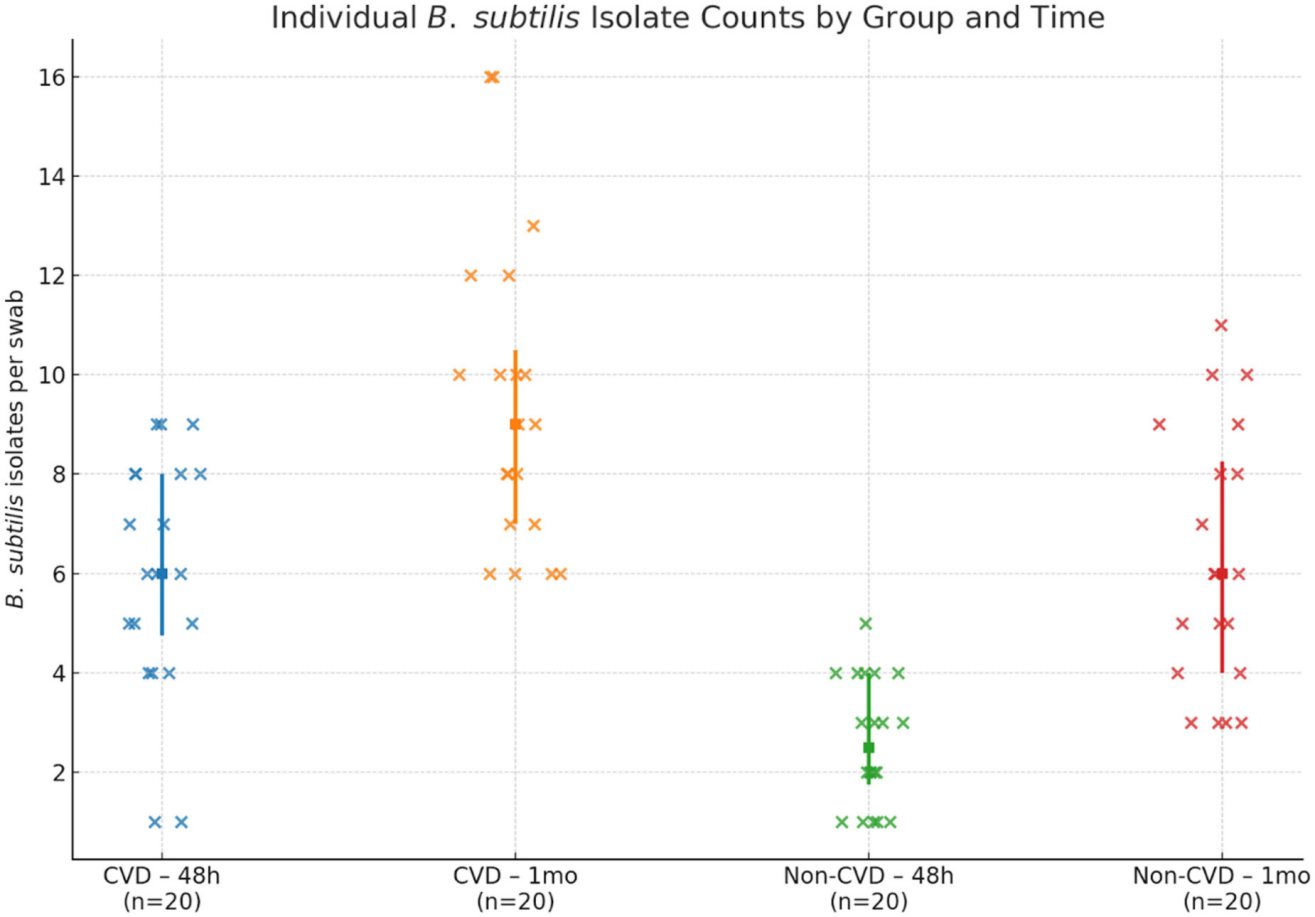
Number of *B. subtilis* isolates recovered from third molar exudate sites at the time of extraction and 1 month after extraction in men with CVD (*n* = 20) and men without CVD (*n* = 20).

The number of *B. subtilis* isolates differed significantly between the CVD group and the non-CVD group at both sampling time points (Figure 5). At the time of surgical extraction, the CVD group exhibited a mean of 6.0 isolates, while the non-CVD group showed a mean of 2.6 isolates, representing a difference of 3.40 isolates between the groups.

The increased number of *B. subtilis* represents the most common infection in postextraction sites in CVD patients (15, 21) and is linked to oral microbiota and systemic cardiovascular health. Some researchers think this happens because oral bacteria slip into the bloodstream and kick off inflammation throughout the body. In our study, we noticed the same trend when we looked at people with cardiovascular disease (CVD) compared to those without. We tracked third molar exudate samples and saw that *B. subtilis* dropped off over time in the non-CVD group. But in people with CVD, B. subtilis kept showing up throughout the 30-day period. Why does it stick around in CVD patients? Lower oxygen, shifts in available nutrients, and the ongoing inflammation in CVD seem to create an environment where spore-forming bacteria like *B. subtilis* can thrive and multiply (22, 23). This observation is attributed to the association with cardiovascular pathology, potentially mediated by altered immune responses in CVD patients resulting from microvascular damage in oral tissues or systemic inflammation (24).

### 3.3 Observation of endospores using light microscopy and TEM

*B. subtilis* forms endospores that can be visualized using light microscopy (Figure 4B). TEM, which provides much higher resolution than light microscopy, enables detailed visualization of endospore structure. Figure 4C presents the typical evaluation of the endospore architecture, as documented before (20, 32), comprising a core (co), a biomembrane (cm), a surrounding cortex (cx), and a multilayered coat (ct).

### 3.4 Investigation of biofilms and their observation using SEM

**Figure 6:**
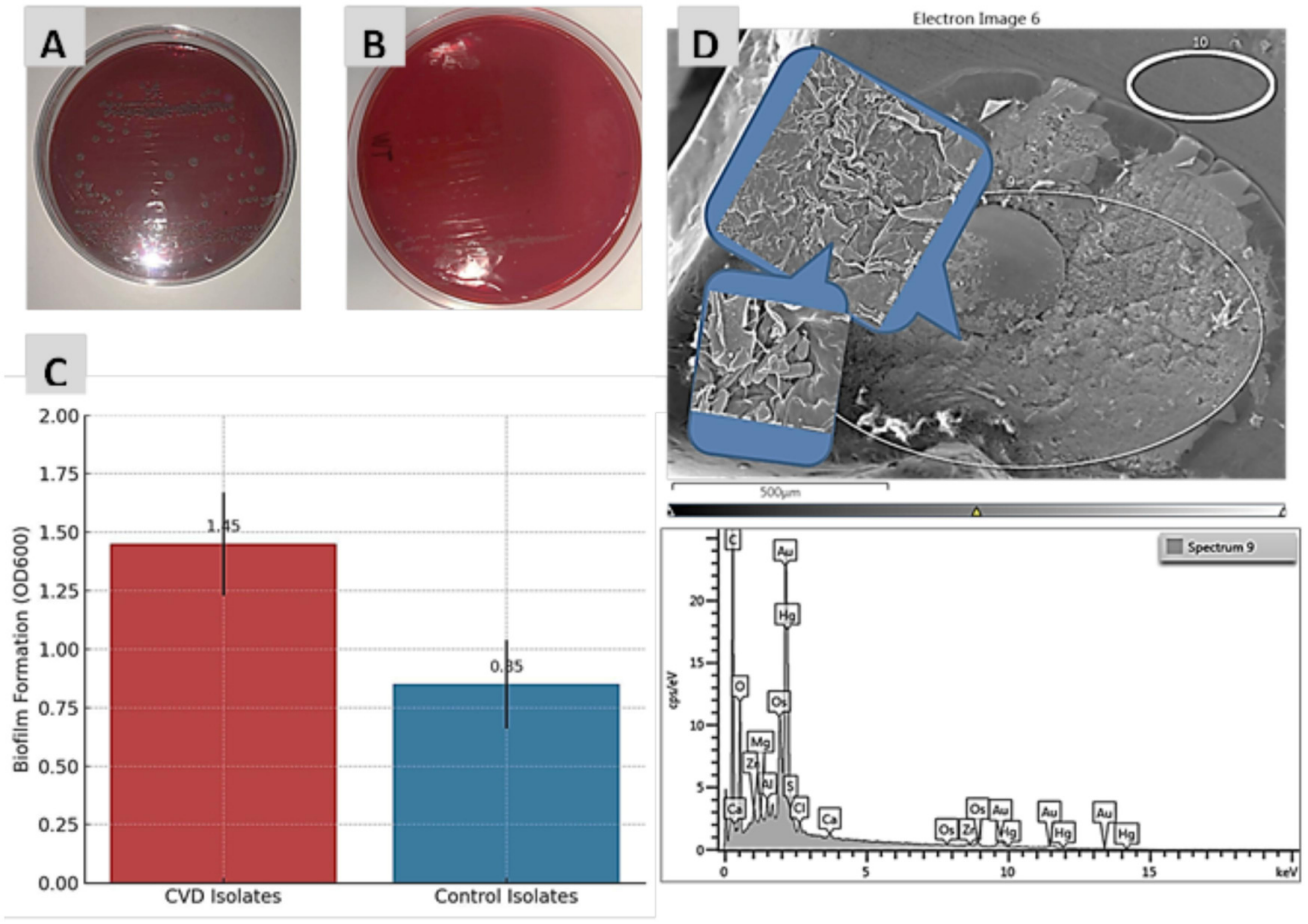
**(A)** Biofilm-forming *B. subtilis* colonies on CRA showing black pigmentation. **(B)** Non-biofilm-forming colonies on CRA showing red pigmentation. **(C)** Percentage of biofilm formation (OD_600_) in *B. subtilis* isolates from CVD patients and control participants. **(D)** SEM micrograph showing biofilm architecture of *B. subtilis* grown on CRA.

Qualitative analysis of biofilms on Congo red agar (CRA) showed that black colonies produced high levels of extracellular polymeric substances (EPS), a matrix composed of polysaccharides, proteins, and nucleic acids that embeds and protects bacterial cells. The EPS component β-1,6-linked *N*-acetylglucosamine binds to Congo red, resulting in dark pigmentation and confirming biofilm formation in some isolates (Figure 6A). In contrast, a limited number of isolates exhibited weak or absent pigmentation due to insufficient EPS production, which prevented effective binding of the Congo red dye (Figure 6B). Biofilm formation was significantly higher (OD_600_) in *B. subtilis* isolates from CVD patients compared to control isolates (Figure 6C). SEM images showed *B. subtilis* biofilms at different magnifications (Figure 6D), confirming the structure and morphology of the EPS produced by biofilm-forming bacteria, as reported recently (20).

### 3.5 Correlations of *B. subtilis* isolates from CVD patients and controls

**Figure 7:**
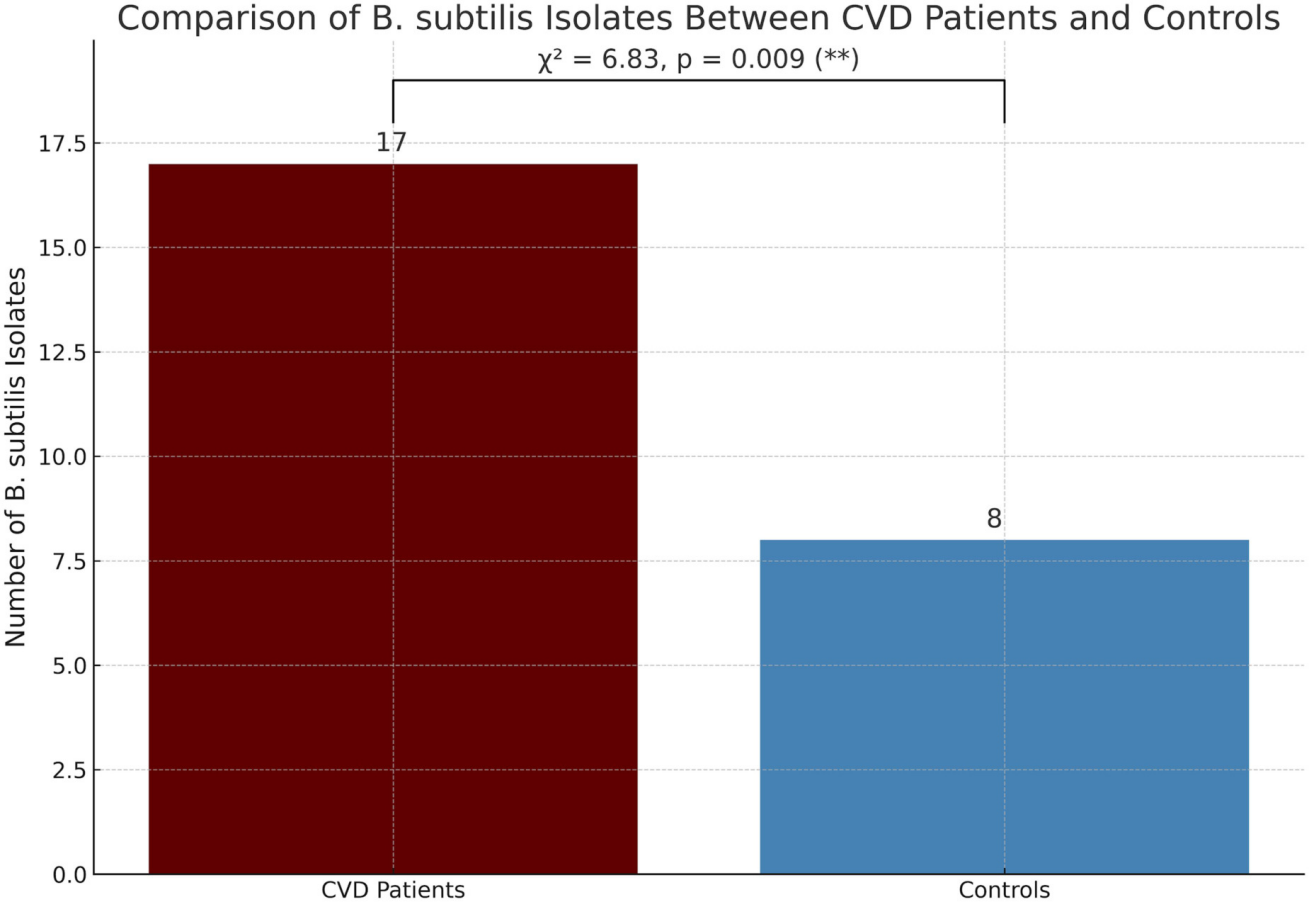
Correlation between *B. subtilis* isolates from CVD patients and control participants.

A potential correlation was observed between the presence of *B. subtilis* (a spore-forming bacterium) in third molar exudates and CVD. As illustrated in Figure 7, a chi-square test showed a statistically significant association between *B. subtilis* colonization and CVD (*χ*² = 6.83, *p* = 0.009). Specifically, 85% (17/20) of CVD patients were carriers of *B. subtilis*, which was significantly greater than the prevalence found in the control group (8/20; 40%).

Although the prevalence of *B. subtilis* in patients with CVD remains largely unknown, numerous other studies have shown the presence of various kinds of bacteria in the blood of CVD patients. For example, a study performed in Madurai, India (25) reported that 47 out of 133 CVD patients (35%) had positive blood cultures, from which 57 bacterial strains were isolated. The predominant species were coagulase-negative staphylococci (CoNS), although other bacterial species were also found.

Kissinger (26) proposed the hypothesis that bacterial infections can damage artery walls in a manner similar to *Helicobacter pylori*, which causes peptic ulcers, suggesting that bacteria can erode the arterial lining and thereby contribute to atherosclerosis. This perspective outlines the potential role of bacterial infection in the etiology of cardiovascular disease, extending beyond traditional models that focus solely on cholesterol levels (27, 28). Therefore, further research is warranted to elucidate the infection-based mechanisms underlying CVD, particularly in light of emerging evidence indicating that the oral microbiome plays an important role in cardiovascular health. *B. subtilis* is commonly found in the oral cavity and has been studied for its probiotic potential. However, its role within the oral atmosphere and its possible impact on CVD biomarkers warrant further investigation. The imaginary mechanical pathways connecting *B. subtilis* to cardiovascular biomarkers involve microbial physiology, host immune reactions, and clinical correlations. *B. subtilis* is known for its strong biofilm-forming ability, which may contribute to periodontal disease under conditions of oral dysbiosis (29).

### 3.6 Association between *B. subtilis* isolation and blood pressure

**Figure 8:**
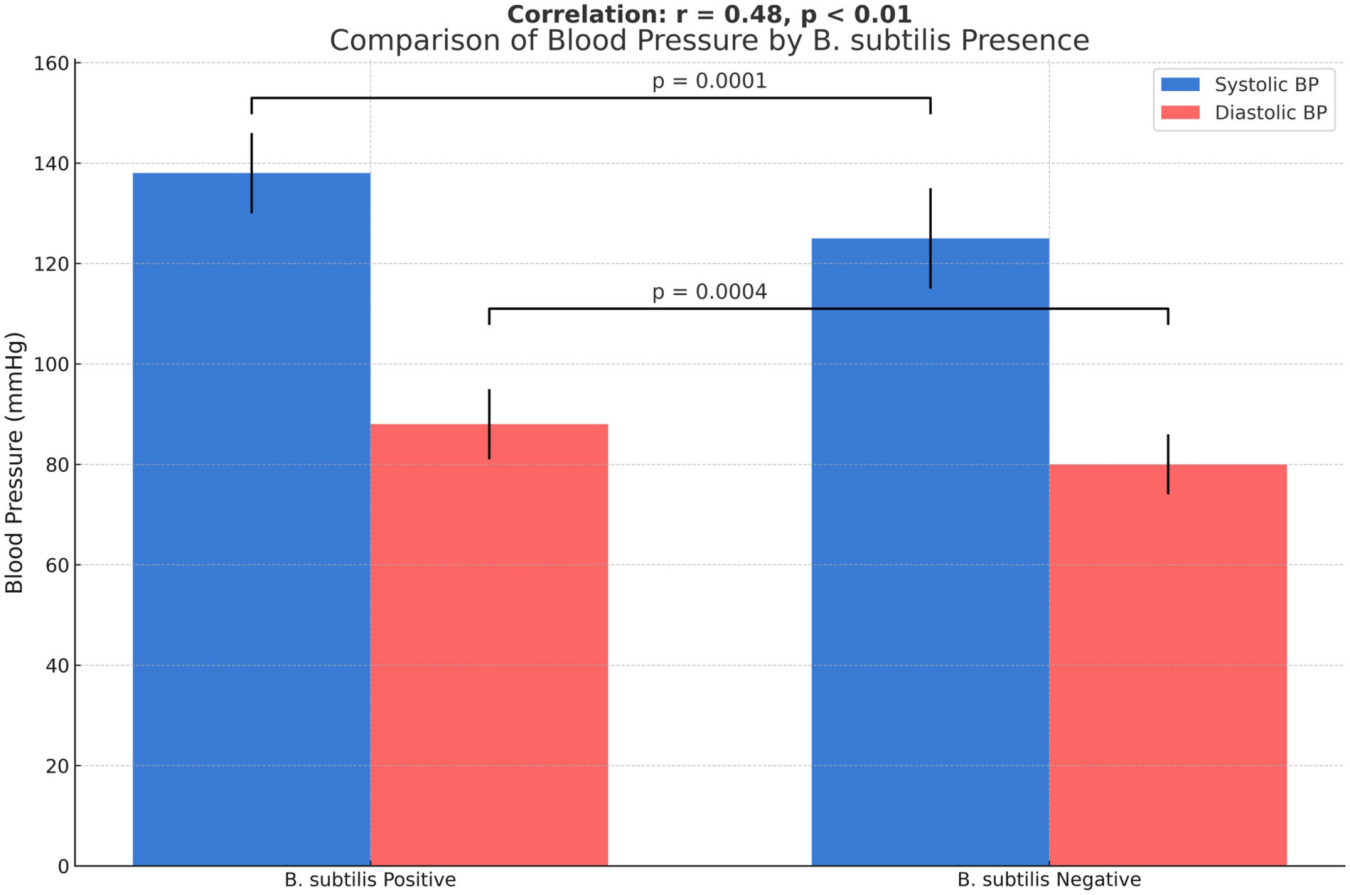
Association between *B. subtilis* isolation and blood pressure.

As shown in Figure 8, group A (patients with *B. subtilis*-positive exudate) exhibited significantly higher systolic blood pressure than group B (*B. subtilis*–negative patients) (*P* < 0.01). Diastolic BP was also significantly higher in group A than in group B (*P* < 0.05). These findings indicate a potential association between the presence of spore-forming *B. subtilis* and an increased risk of cardiovascular disease, possibly mediated by low-grade systemic inflammation or microbiome-related mechanisms. The data indicate that the *B. subtilis*-positive group showed a particularly high systolic and diastolic blood pressure, which supports a potential association with increased cardiovascular disease risk. A moderate positive correlation was observed between the presence of *B. subtilis* and elevated blood pressure (*r* = 0.48, *P* < 0.01).

### 3.7 Mechanistic pathways linking oral *B. subtilis* to CVD

**Figure 9:**
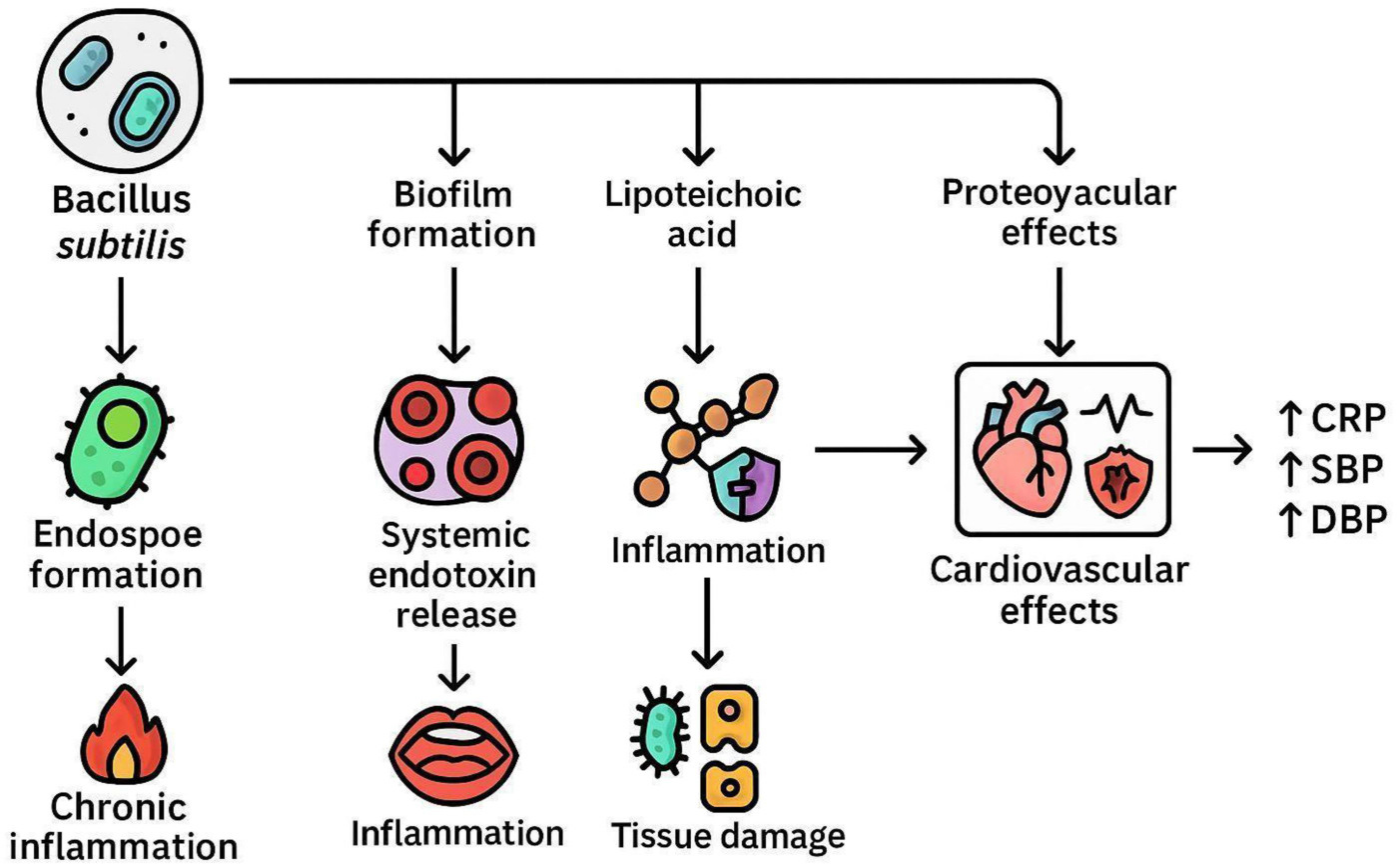
Imaginary mechanistic pathways linking oral *B. subtilis* to CVD. CRP refers to C-reactive protein, a marker of inflammation whose elevated levels are associated with increased risk of coronary disease. SBP denotes systolic blood pressure, representing the highest value during hypertensive measurement; it 's a measure of pressure during active cardiac contraction. DBP denotes diastolic blood pressure, which reflects the following value during hypertensive Reading. SBP shows the pressure in your arteries when the heart contracts with each heartbeat. This surge in pressure happens at the peak of the heart's pumping cycle.

As shown in the mechanism systematic framework (Figure 9), oral pathogens (*B. subtilis*) and their biofilms may be associated with CVD through a key risk factor (systemic inflammation). Chronic oral infections can elevate systemic inflammatory markers such as C-reactive protein (CRP), interleukin-6 (IL-6), and tumor necrosis factor-alpha (TNF-α), all of which are predictors of CVD risk.

This association may occur through the translocation of *B. subtilis* into the bloodstream via a compromised oral mucosa during activities such as tooth brushing or dental procedures. Once in the bloodstream and eventually into circulation, these bacteria can trigger systemic inflammatory reactions that contribute to endothelial dysfunction and atherogenesis, potentially mediated by structural components such as lipoteichoic acid (LTA) (30). The progression of CVD may be driven by interactions between LTA and Toll-like receptor 2 (TLR2) on host immune cells, which can lead to the activation of pro-inflammatory signaling pathways (3). This immune system activation leads to the release of cytokines and other inflammatory mediators, which contribute to vascular inflammation and plaque formation. Previous studies have shown that LTA derived from *B. subtilis* induces NF-INB activation in cells expressing TLR2, highlighting the role of TLR2 in recognizing Gram-positive bacterial components. *B. subtilis* can contribute to systemic inflammation that adversely affects lipid metabolism and promotes atherogenesis. Animal studies have shown that polysaccharides derived from *B. subtilis* can reduce total cholesterol, LDL, VLDL, and triglycerides by increasing HDL levels (31).

### 3.8 Limitations of the study

This study has some limitations that must be considered, such as restrictions related to participant gender (female) and tooth position. Further genomic characterization of the *B. subtilis* isolates could clarify whether strains recovered from CVD patients harbor genetic determinants related to pathogenicity or proinflammatory signaling. The exclusion of some patients based on some criteria may limit the generalizability of the findings to broader populations.

## 4 Conclusion

In developing countries such as Iraq, the absence of NHS dental treatment limits researchers from optimizing oral treatment strategies based on patient-derived data to investigate associations between oral conditions and other diseases. Therefore, this study provides a protocol for conducting investigations related to oral health through collaboration with other institutions, such as universities. Regarding the main finding of this study, CVD patients showed a higher prevalence of *B. subtilis* in their third molar exudates compared to healthy controls. *B. subtilis* was found more frequently in third molar exudates from patients with CVD compared with healthy controls. This finding supports the hypothesis that the oral microbiota, including spore-forming bacteria, may play a role in the progression of cardiovascular health and disease. The imaginary route mentioned in this study shows that *B. subtilis* can affect cardiovascular biomarkers through biofilm formation, immune activation, and systemic inflammation. Further research is required to clarify these relationships and to determine the clinical significance of *B. subtilis* in oral and cardiovascular health.

## Data Availability

The original contributions presented in the study are included in the article/Supplementary Material; further inquiries can be directed to the corresponding author/s.
